# Influence of fermented milk permeate containing antimicrobial *Lactobacillus* and galactooligosaccharides on growth performance and health parameters in neonatal piglets

**DOI:** 10.3389/fvets.2025.1501117

**Published:** 2025-02-20

**Authors:** Sarunas Badaras, Vytaute Starkute, Ernestas Mockus, Modestas Ruzauskas, Dovile Klupsaite, Erika Mozuriene, Jurgita Dailidaviciene, Agila Dauksiene, Laurynas Vadopalas, Elena Bartkiene

**Affiliations:** ^1^Institute of Animal Rearing Technologies, Faculty of Animal Sciences, Lithuanian University of Health Sciences, Kaunas, Lithuania; ^2^Department of Food Safety and Quality, Faculty of Veterinary Medicine, Lithuanian University of Health Sciences, Kaunas, Lithuania; ^3^Department of Anatomy and Physiology, Faculty of Veterinary Medicine, Lithuanian University of Health Sciences, Kaunas, Lithuania; ^4^Institute of Microbiology and Virology, Faculty of Veterinary Medicine, Lithuanian University of Health Sciences, Kaunas, Lithuania

**Keywords:** metataxanomic, by-products bioconversion, immunoglobulins, volatile compounds, antimicrobials

## Abstract

The study aimed to compare the effects of fermented milk permeate (MP) containing *Pediococcus pentosaceus* (MPPp) and *P. acidilactici* (MPPa) on growth performance, plasma parameters, and the faecal microbial, metataxonomic, and physicochemical characteristics of Topigs Norsvin Yorkshire piglets. A total of 36 1-day-old piglets were divided into three groups: (i) control group (C), (ii) MPPp group, and (iii) MPPa group. The treated groups, in addition to their full-fledged combined pre-starter diet, received 25 mL of MP daily. After the experiment, piglets in the MPPa group exhibited the highest weight gain, while piglets in the MPPp group showed the highest IgM concentration. Both experimental groups demonstrated increased *Lactobacillus* counts in the faeces. Although the numbers of *Lactobacillus* and *Enterobacteria* increased, these microbial changes did not show a direct correlation with growth performance. The feces of MPPa piglets had a unique volatile compound profile, characterized by higher levels of butanoic acid and indole levels, which may be linked to differences in their metataxonomic profile. The MPPp group showed a greater variety of bacterial patterns compared to the control and MPPa groups. Post-experiment, the MPPa group demonstrated the highest prevalence of specific bacterial species, *Parabacteroides* sp. 12,306, *Terrisporobacter* sp. 34,393, *Holdemanella* sp. 36,738, and *Lachnospiraceae* sp. In conclusion, feeding piglets with MPPa proved beneficial for achieving better weight gain while also promoting the proliferation of specific bacteria species and contributing to a distinctive VC profile in their faeces. These findings highlight the importance of further research into the metabolic pathways underlying these observations.

## 1 Introduction

After birth, newborn animals enter a farm environment, which is naturally rich in various microorganisms, including opportunistic pathogens. Newborn and young animals’ immune systems are not fully developed and are at a higher risk of disease than older ones (1). Additionally, The spread of antibiotic-resistant pathogens underscores the need to reduce antibiotic use in pig farming and prioritize strategies that promote beneficial gut bacteria from birth. These challenges are driving research into alternative dietary interventions for commercial pig farms. The majority of studies have analysed postweaning dietary manipulation as well as maternal dietary intervention (2–6). Therefore, dietary intervention for newborn piglets could also be a promising alternative to improve animal health status from the first days of life.

In this study, we hypothesised that fermented milk permeate (MP) could be a valuable feed material for newborn piglets, as it possesses antimicrobial properties, contains a high number of viable lactic acid bacteria (LAB), and includes galactooligosaccharides (GOS) with prebiotic effects (7). MP is a secondary product of milk protein production obtained by membrane fractionation of milk. It is a biosafe that contains lactose, minerals, and serum proteins. Previous studies demonstrated that specific LAB strains can convert lactose into GOS during MP fermentation (7). Additionally, fermented MP possesses desirable antimicrobial properties against various pathogenic and opportunistic strains. Therefore, feeding such an additional feed material to piglets from the 1st day of their life can have a multifunctional effect: probiotics – due to a high number of viable LAB. Prebiotics are due to GOS; antimicrobials are due to organic acids and other LAB metabolites in fermented MP.

Probiotics can act as the host directly and indirectly via the production of postbiotics. The reduction of environmental pH is a desirable change because inhibiting a range of non-desirable microorganisms, however, is conducive to good ones (8, 9). It was reported that certain probiotic bacteria can control the proliferation of pathogenic bacteria and significantly modulate the gastrointestinal tract microbiota (10). Prebiotics enhance beneficial microbes’ proliferation and abundance, increasing the production of desirable metabolites and suppressing the proliferation of pathogens (11).

It was reported that, due to the high plasticity of the young piglets’ gastrointestinal tract microbiota, promoting it at the early stage of life presents the most effective opportunity to improve animal health (2, 12). Rapid initial colonisation of the population of the gastrointestinal tract microbiota is termed the “pioneer microbiota” (13). It was reported that the pioneer microbiota plays an important role in the functioning of the immune system at the later stages of animal life (14).

Another important strategy in pig farms is to detect sensitive markers that indicate the animal’s health status. It was reported that 12.3% of piglets cannot survive before weaning (15). Our other hypothesis is that the changes in the volatile compounds (VC) profile of the piglets’ faeces can be related to the changes (desirable as well as non-desirable ones) of the piglets’ gastrointestinal tract microbial community, and the latter changes can be associated with the animal health status and growth performance. Faeces VC profile can provide information about the status of the piglets’ health and welfare, followed by timely applied prevention practice, thereby prolonging the piglets’ lives. Information about the faeces’ VC profile and VC relations with the other piglet’s health status characteristics can be useful in the future for more precision livestock farming organisations.

Taking into consideration that gastrointestinal tract microbiota plays a key role in feed efficiency (16), growth performance (17), and disease (18) in pigs, the testing of new sustainable feed materials with the aim of targeting improvements in the GIT microbiota can be very promising.

The study aimed to compare the effects of fermented milk permeate (MP) with *Pediococcus pentosaceus* (MPPp) and *P. acidilactici* (MPPa) on growth and plasma parameters, faecal microbial, metataxonomic, and physicochemical characteristics in Topigs Norsvin Yorkshire piglets. Moreover, the faecal VC profile was analysed as a possible chemical marker related to changes in animal health and growth performance characteristics.

## 2 Materials and methods

### 2.1 Fermented milk permeate

Milk permeate was obtained from the agricultural cooperative “Pienas LT” (Biruliskes, Lithuania) and stored at −18°C before fermentation. Zokaityte et al. (7) reported characteristics of the fermented MP, which are given in Supplementary material S1 Characteristics of the fermented milk permeate (Supplementary Tables S1–S3).

### 2.2 Animals’ housing and experimental design

The study was conducted on a pig breeding farm in the Klaipeda district (Vanagai, Lithuania) and the Institute of Animal Rearing Technologies, Lithuanian University of Health Sciences (Kaunas, Lithuania). The experiment followed the guidelines outlined in the Republic of Lithuania Act (19). A 25-day experiment was conducted using 36 1-day-old Topigs Norsvin Yorkshire piglets, with 12 piglets assigned to each group. Piglets were selected from three litters of third-parous sows with very similar reproductive performance. The 36 piglets were mixed and allocated to each group based on their weight to ensure uniformity. Each group included three smaller, six medium, and three larger piglets housed in farrowing pens with third-parous sows. Three dietary treatments were compared: (i) C – control group, after day 7th fed with full-fledged combined pre-starter feed for piglets PANTO® pre (Hamburger Leistungsfutter GmbH, Hamburg, Germany); treated groups, from the 1st day of life and, additionally to the full-fledged combined pre-starter feed for piglets, after the 7th day of life, received 25 mL of fermented MP: (ii) group MPPp received MP fermented with *P. pentosaceus* strain; (iii) group MPPa received MP fermented with *P. acidilactici* strain. Wet feed was prepared by diluting the feed with water (ratio 1:3), and, for both treated groups, for each piglet, additionally, 25 mL of fermented MP was added to a water–feed mixture. The farrowing crates (containing a heated creep mat for piglets) were 4.0 m^2^ (1.6 m × 2.5 m), of which 0.20 m^2^ was for piglets and 1.50 m^2^ was for sows. The groups were formed on an analogue basis. The sows were fed with compound feed (in accordance with NRC requirements) (20). Drinking water was available *ad libitum* throughout the trial. The trial started with piglets at an initial body weight of 1.56 kg in all (control and both treatment) groups.

**Figure 1:**
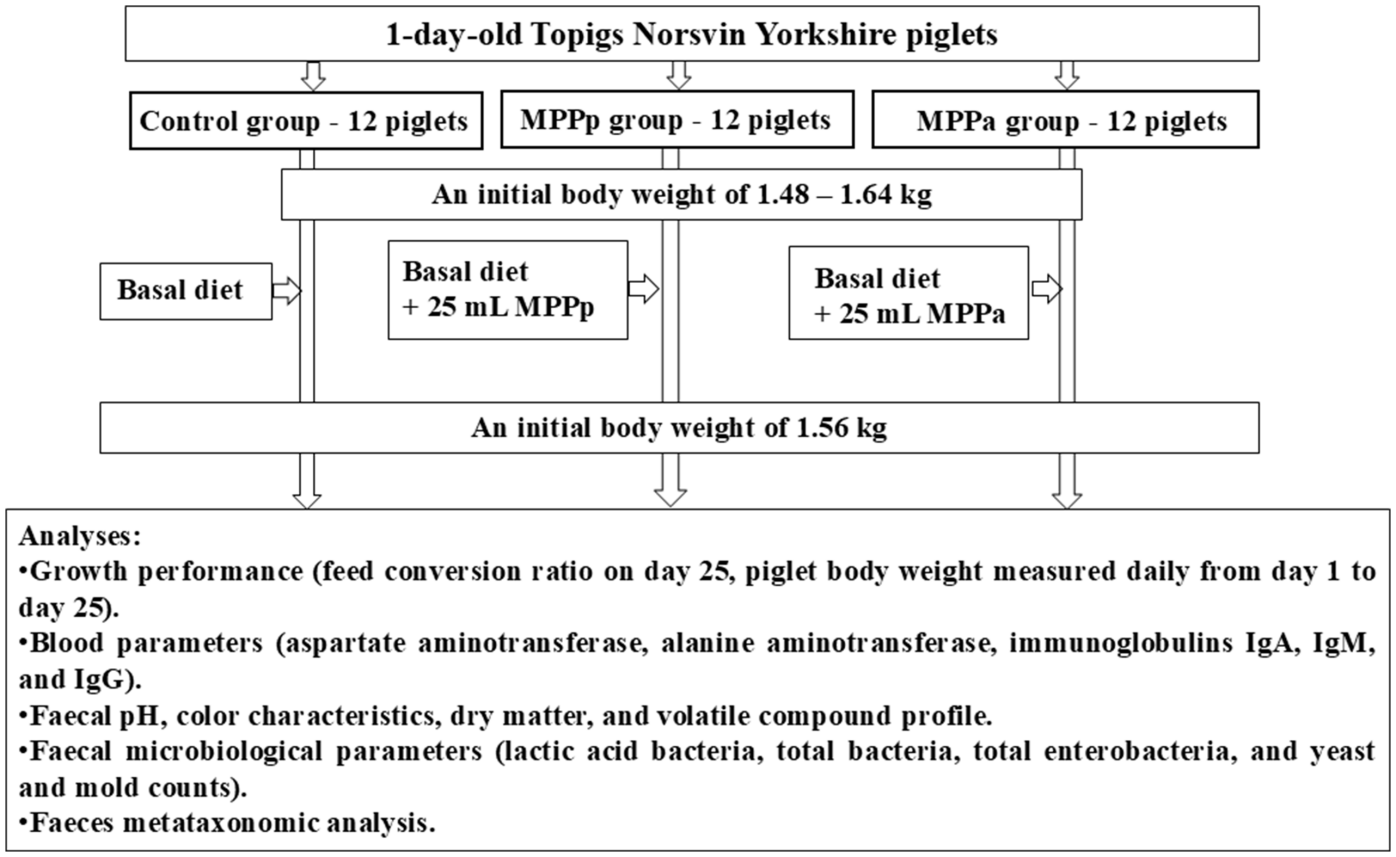
The principal scheme of the experiment (MPPp – treated piglets group fed a basal diet supplemented with 25 mL/day fermented with *Pediococcus pentosaceus* milk permeate; MPPa – treated piglets group fed a basal diet and supplemented with 25 mL/day fermented with *Pediococcus acidilactici* milk permeate).

The piglet’s diet before the trial consisted of 17.5% crude protein, 4.20% crude fibre, 10.8% crude fats, 1.45% av. lysine, 0.55% av. methionine, 0.60% Ca, and 0.60% av. P. No antibiotic treatment was administered. The principal scheme of the experiment is shown in Figure 1.

The basal feed was formulated according to the nutritional requirements described in (20). The basal feed composition and nutritional value are shown in Table 1. Dietary contents were analysed according to the AOAC recommendations (21).

**Table 1:** Composition of control and experimental piglet diets.

Ingredients	Content (%)
Wheat (soaked)	21
Barley	12
Linseed, hydrothermally treated	9.9
Soybeans steam-heated	7
Wheat bran	7
Whey powder	5
Oat flakes	5
Soybean meal, with stock, steam-heated	5
Sugar	3.8
Barley, soaked	3
Soy protein concentrate	2.5
Monocalcium phosphate	1.1
Whey permeate	1
Calcium carbonate	1
Rapeseed oil	1
Yeast	0.5
Potato protein	0.5
Sodium chloride	0.5
Vegetable oil (coconut, palm)	0.4
Product from lignite, rich in humic acid	0.2
Mono- and diglycerides of fatty acids	0.2
Magnesium oxide	0.1
Premix, amino acids, and other additives	12.3
Nutritional value	
ME swine (MJ/kg)	14.4
Crude protein (%)	17.5
Crude fat (%)	10.8
Crude fibre (%)	4.2
Lysine (%)	1.45
Methionine (%)	0.55
Threonine (%)	1.1
Tryptophan (%)	0.4
Methionine + Cystine (%)	1.0
Ca (%)	0.60
Total P (%)	0.60
Available P (%)	0.4
Na (%)	0.25

Composition of premix per 1 kg of feed: 16000 IU vitamin A; 2000 IU vitamin D3; 170 mg vitamin E; 30 mg iron as ferrous sulfate monohydrate; 200 mg iron as ferrous fumarate; 130 mg copper as dicopper chloride trihydroxide; 80 mg zinc as zinc chloride hydroxide monohydrate; 60 mg manganese as manganese (II) oxide; 1.2 mg iodine as calcium iodate, anhydrous; 0.4 mg selenium as sodium selenite. For all groups, the following were added to the compound feed: 613 FYT 6-phytase EC 3.1.3.26; 2,800 XU endo-1,4-ß-xylanase EC 3.2.1.8; 1,609 BGU Endo-1,3(4)-ßglucanase EC 3.2.1.6; 60 PGLU polygalacturonase EC 3.2.1.15; 5,000 mg benzoic acid. Technological additives: 2290 mg formic acid; 2040 mg formic acid from calcium formate; 1,100 mg lactic acid from calcium lactate; 890 mg propionic acid; 510 mg citric acid; 2.9 mg propyl gallate; 80 mg butylated hydroxytoluene (BHT).

### 2.3 Evaluation of piglets’ growth performance

Individual body weight (BW) gain was recorded every day of age manually with an electronic MS Schippers weighing platform 100 kg scale (model type: PSSST, BOSCHE GmbH & Co. KG, Germany). The feed conversion ratio (FCR) was calculated based on feed intake (87% of dry matter) and BW gain, which was recorded on the same day as BW gain.

### 2.4 Plasma analysis

Only six samples were taken from each group to avoid stressing newborn piglets. Plasma biochemical variables were evaluated on the 2nd and 25th days of the piglets’ lives. Before the morning feeding, piglets were bled from the jugular vein into vacuum blood tubes (BD Vacutainer, United Kingdom). Tubes with clot activators were used for biochemical examination. The parameters included aspartate aminotransferase, alanine aminotransferase, and immunoglobulins IgA, IgM, and IgG, which were analysed with an automatic biochemistry analyser in the accredited laboratory ‘Anteja’ (Klaipeda, Lithuania).

### 2.5 Evaluation of faecal pH, dry matter, and texture hardness

Evaluation of faecal pH and colour characteristics was performed at the end of the experiment because, on the 1st day of piglets’ lives, there was not enough sample to perform the above-mentioned analyses. The faecal pH was analysed with a pH meter (Inolab 3, Hanna Instruments, Italy). The dry matter (DM) of the faeces was evaluated after drying the samples at 103 ± 2°C to a constant weight. Texture hardness was measured as the energy required for sample deformation (CT3 Texture Analyzer, Brookfield, Middleboro, MA, United States).

### 2.6 Microbiological analysis of faecal samples

Faecal samples were collected from 12 piglets from each group before (at day 1) and after (at day 25) the experiment, stored in vials (+4°C) with transport medium (*Faecal Enteric* Plus, Oxoid, Basingstoke, UK) and analysed on the same day. LAB, total enterobacteria (TEC), and yeast/mould (Y/M) counts were evaluated following the methods described by Zavistanaviciute et al. (22).

### 2.7 Metataxonomic analysis of the bacterial composition in the faecal samples of piglets’

After the piglets were divided into groups, faeces from 12 piglets from each group were taken, and a single pooled sample was prepared for microbiome profiling using next-generation targeted sequencing of 16S rRNA. After the experiment, faeces from 12 piglets from the control and both treated groups were collected (36 samples in total), and three pooled samples representing each group were prepared. Samples were stored in DNA/RNA Shield (1:10 dilution; R1100-250, Zymo Research, United States) at −70°C until DNA extraction. ZymoBIOMICS^®^-96 MagBead DNA Kit (Zymo Research, Irvine, CA) was used to extract DNA using an automated platform according to manufacturer instructions. Bacterial 16S ribosomal RNA gene-targeted sequencing was performed using the *Quick*-16S^™^ NGS Library Prep Kit (Zymo Research, Irvine, CA). Primers of the V3–V4 region, 338F (5′-ACTCCTACGGGAGGCAGCAG-3′) and 806R (5′-GGACTA CNNGGGTATCTAAT-3′), were used to amplify 16S rDNA to investigate microbial composition. The sequencing library was prepared using real-time PCR. The final PCR products were quantified with qPCR fluorescence readings and pooled together based on equal molarity. The final pooled library was cleaned with the Select-a-Size DNA Clean & Concentrator^™^ (Zymo Research, Irvine, CA), then quantified with TapeStation^®^ (Agilent Technologies, Santa Clara, CA) and Qubit^®^ (Thermo Fisher Scientific, Waltham, WA). The final library was sequenced on Illumina^®^ Nextseq^™^ with a P1 reagent kit (600 cycles). The sequencing was performed with a 30% PhiX spike-in. Unique amplicon sequence variants were inferred from raw reads using the DADA2 pipeline (23). Potential sequencing errors and chimeric sequences were also removed with the Dada2 pipeline. The taxonomy assignment was performed using Uclust from Qiime v.1.9.1 with the Zymo Research Database, a 16S database that was internally designed and curated as a reference. Sequences were deposited at the NCBI database by the access number PRJNA 1205751. Composition visualisation and alpha-diversity analysis were performed with Qiime v.1.9.1 (24). The number of genome copies per microlitre DNA sample was calculated by dividing the gene copy number by an assumed number of gene copies per genome.

### 2.8 Analysis of the faecal volatile compound profile

Faeces were prepared for gas chromatography (GC) analysis using solid-phase microextraction (SPME). An SPME device with Stableflex (TM) fibre, coated with a 50-μm DVB-PDMS-Carboxen™ layer (Supelco, United States), was used for sample preparation. For gas chromatography–mass spectrometry (GC–MS), a GCMS-QP2010 (Shimadzu, Japan) was used. The gas chromatograph was equipped with an AOC-5000 Plus Shimadzu autosampler, upgraded with an SPME analysis kit. Analysis was performed according to the procedure described by Vadopalas et al. (25).

### 2.9 Statistical analysis

A paired samples t-test was used to compare differences in parameter means between the groups (C, MPPp, MPPa). For growth performance, daily data were collected (*n* = 12 from each group); for plasma parameters, samples were analysed at the beginning and at the end of the experiment (*n* = 6 from each group). The pH and dry matter of piglet faeces were analysed at the end of the experiment (*n* = 12 from each group). Microbiological parameters of faecal samples were evaluated at the beginning and at the end of the experiment (*n* = 12 from each group).

For metataxonomic analysis of bacterial composition in the faecal samples, samples were collected from 12 piglets per group at the beginning of the experiment, with a single pooled sample prepared for microbiome profiling. At the end of the experiment, faecal samples from 12 piglets from each group (C, MPPp, and MPPa) were collected, and three pooled samples (one per group) were prepared and analysed. For the faecal sample VC profile, individual analysis was conducted on 12 samples from 36 piglets at the beginning of the experiment, and at the end, 12 samples from each group were analysed. The influence of diet was determined using multivariate tests of between-subjects effects, with baseline measurements used as covariates to account for experimental conditions. Mean values were compared using Duncan’s multiple range *post hoc* test, with significance set at a *p-*value of ≤0.05. Results are presented in the tables as mean values with pooled standard errors. Additionally, Pearson’s correlations between characteristics were calculated, and the strength of correlations was interpreted following Evans (26).

Correlations were deemed significant at a *p*-value of *≤* 0.05. Differences in bacterial genera/species between the groups were assessed using the *Z*-test calculator for two population proportions (27). The number of reads for each genus/species was counted from the total reads in the samples, and the relative abundance was compared between the groups. A two-tailed hypothesis was applied.

A standard (D6300, Zymo Research, Murphy Ave., Irvine, CA, United States) of mixed known bacterial cultures was sequenced alongside sample sequencing for the quality control of taxonomical identification. Statistical comparisons were considered significant at a *p-*value *of* ≤ 0.05. Heatmap visualisation and analysis were conducted using the R statistical programming software package “ComplexHeatmap” (version 2.14.0). Partial least squares discriminant analysis (PLS-DA) and variable importance of projection (VIP) analyses were conducted using the “mixOmics” package (version 6.22.0). Volcano plots were generated with the “EnhancedVolcano” package (version 1.16.0).

## 3 Results

### 3.1 Piglets’ growth performance

**Figure 2:**
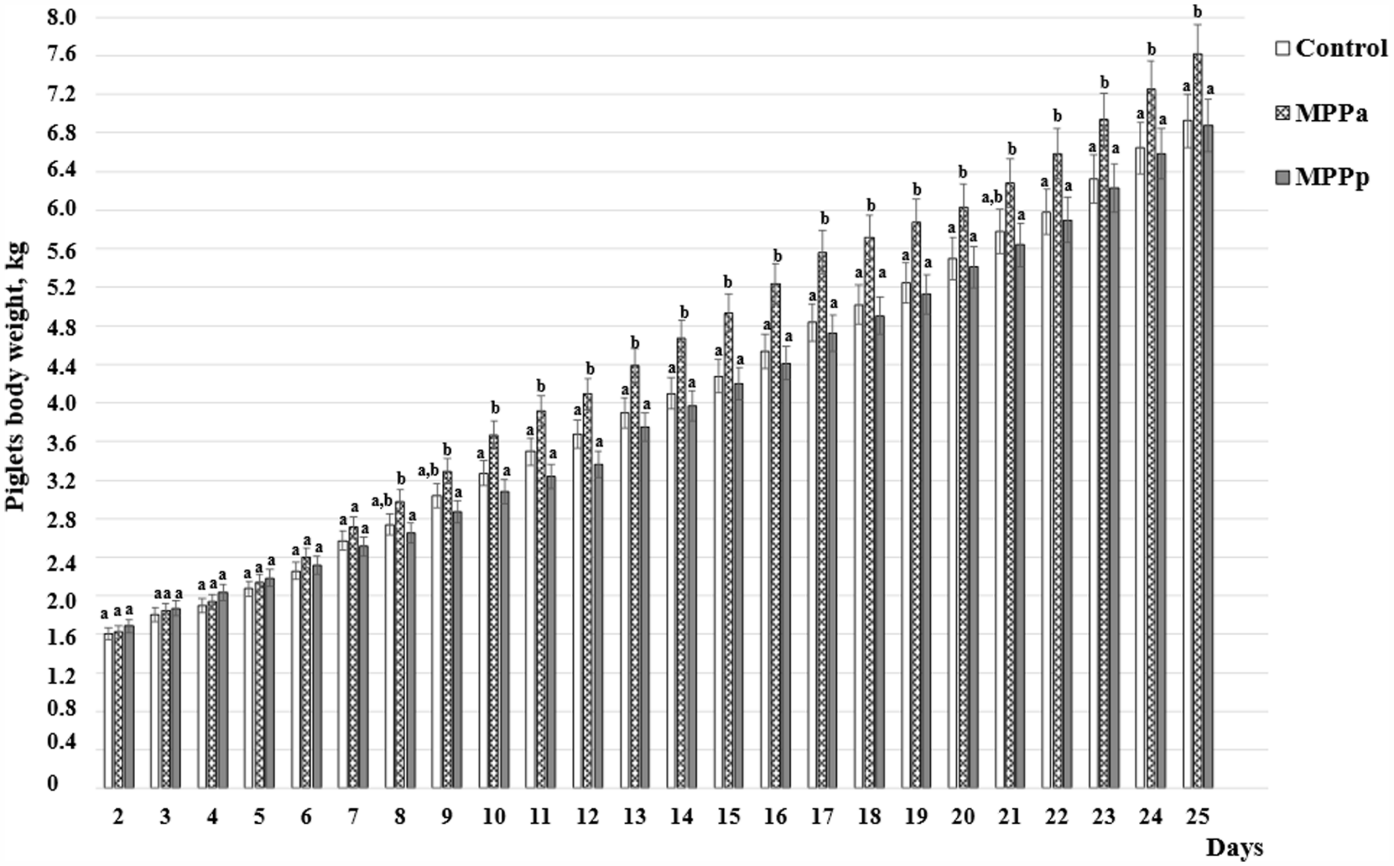
Piglets’ body weight, kg (C – control group fed a basal diet, MPPp – treated group fed a basal diet and supplemented with 25 mL/day fermented with *Pediococcus pentosaceus* milk permeate; MPPa – treated group fed a basal diet and supplemented with 25 mL/day fermented with *Pediococcus acidilactici* milk permeate). ^a,b^ Different letters indicate significant differences among different piglet groups on the same day (*p* ≤ 0.05). The data are presented as the mean ± standard deviation (*n* = 12/group).

The piglets’ body weight, measured every day of their life, is shown in Figure 2. As could be seen from the data, despite that, significant differences in their body weight were not found from the 1st to the 7th day of piglets’ lives. From the 8th day of piglets’ lives till the end of the experiment, the highest body weight showed MPPa group piglets compared to the control and MPPp groups. Significant body weight differences were not established throughout the whole experiment period between the control and MPPp group piglets.

**Figure 3:**
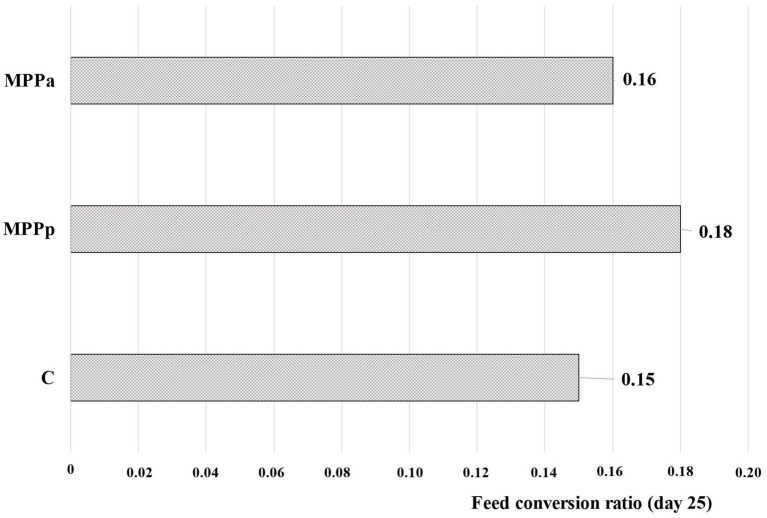
Feed conversion ratio calculated on day 25 (C – control group fed a basal diet, MPPp – treated group fed a basal diet supplemented with 25 mL/day fermented with *Pediococcus pentosaceus* milk permeate; MPPa – treated group fed a basal diet supplemented with 25 mL/day fermented with *Pediococcus acidilactici* milk permeate).

The highest feed conversion ratio (0.18, calculated on day 25) showed the MPPp group (Figure 3). Control and MPPa group’s feed conversion ratios, in comparison with MPPp, were found to be lower (0.15 and 0.16, respectively). Finally, further studies were performed to identify differences in piglet plasma and faecal parameters and their possible relations with the growth performance in different animal groups.

### 3.2 Piglets plasma parameters

Plasma parameters of 1- and 25-day-old piglets are shown in Table 2. In comparison, IgA concentration in all groups at the beginning and the end of the experiment was <0.33. In comparison, IgM concentration in piglets’ plasma at the beginning of the experiment, significantly lower IgM concentration in MPPa piglets’ group plasma was found compared with control and MPPp groups. At the end of the experiment, the highest IgM concentration showed MPPp group piglet plasma samples, and the diet was a significant factor in IgM concentration in piglet plasma (*p* = 0.046). At the experiment’s beginning and end, the differences in IgG concentration between the different group samples were not established. However, in comparison, IgG concentration at the beginning and the end of the experiment, at the end of the experiment, IgG concentration in control, MPPp, and MPPa groups plasma samples were, on average, 7.30, 7.34, and 6.24 times, respectively, lower. Significant differences between the concentration of alanine aminotransferase and aspartate aminotransferase at the experiment’s beginning and end were not found, and the diet was not a significant factor in these plasma parameters.

**Table 2:** Plasma parameters of 1-day-old and 25-day-old piglets.

Plasma parameters		Treatments			*p*			
		C	MPPp	MPPa	C × MPPp	C × MPPa	MPPp × MPPa	Significance of the analysed factor (diet)
Immunoglobulin (IgA), g/L	1 day	<0.33	<0.33	<0.33	-	-	-	-
	25th day	<0.33	<0.33	<0.33	-	-	-	-
Immunoglobulin (IgM), g/L	1 day	0.387 ± 0.151^b^	0.525 ± 0.119^a^	0.213 ± 0.008^a^	0.175	**0.041**	**0.001**	-
	25th day	0.225 ± 0.028^a^	0.380 ± 0.144^a^	0.285 ± 0.086^a^	**0.037**	0.157	**0.042**	**0.046**
Immunoglobulin (IgG), g/L	1 day	15.4 ± 3.16^b^	14.9 ± 7.08^b^	11.1 ± 1.57^b^	0.903	0.057	0.218	-
	25th day	2.11 ± 0.281^a^	2.03 ± 0.447^a^	1.78 ± 0.613^a^	0.743	0.281	0.565	0.462
ALT, U/L	1 day	58.8 ± 8.98^a^	59.5 ± 10.6^b^	56.7 ± 10.7^a^	0.543	0.746	0.679	-
	25th day	52.0 ± 12.3^a^	46.7 ± 6.06^a^	44.2 ± 7.11^a^	0.429	0.277	0.286	0.326
AST, U/L	1 day	143.7 ± 128.1a	107.0 ± 34.2^b^	150.0 ± 58.3^b^	0.529	0.935	0.124	-
	25th day	31.7 ± 10.6^a^	35.2 ± 6.05^a^	30.7 ± 4.68^a^	0.609	0.855	0.194	0.568

C – control group fed with basal diet, MPPp – treated group fed a basal diet supplemented with 25 mL/day fermented with *Pediococcus pentosaceus* milk permeate; MPPa – treated group fed a basal diet supplemented with 25 mL/day fermented with *Pediococcus acidilactici* milk permeate. ALT – Alanine aminotransferase; AST – Aspartate aminotransferase; U – Units; 1 day – at the beginning of the experiment; 25th day – at the end of the experiment. ^a,b^ different letters indicate significant time-related differences (*p* ≤ 0.05). The data are presented as the mean ± standard deviation (*n* = 6/group). Bold values indicate significant difference between tested treatments or significant effect of the analyzed factor on tested parameters, when *p* ≤ 0.05.

### 3.3 Piglet faecal pH, dry matter, texture hardness, and colour characteristics

The pH, dry matter, and texture hardness of piglet faeces at the end of the experiment (on the 25th day of life) are shown in Table 3. Significant differences between the faeces’ pH at the end of the experiment were not found, and the diet was not a significant factor in the faeces’ pH. However, significantly higher dry matter content and, on the opposite end, significantly lower faeces texture hardness were found in both treated groups in comparison with the control samples. A moderate negative correlation was established between faeces texture hardness and dry matter (*r* = −0.471, *p* = 0.004).

**Table 3:** The pH, dry matter, and texture hardness of piglet faeces at the end of the experiment (25th day of life).

Faecal parameters	C	MPPp	MPPa	*p*			
				C × MPPp	C × MPPa	MPPp×MPPa	Significance of the analysed factor (diet)
	On the 25^th^ day of life						
pH	6.38 ± 0.75	6.35 ± 0.850	6.47 ± 0.78	0.945	0.775	0.726	0.929
Dry matter (%)	33.6 ± 6.76	46.5 ± 12.6	44.36 ± 14.6	**0.005**	**0.033**	0.723	**0.025**
Texture hardness (mJ)	0.420 ± 0.070	0.210 ± 0.030	0.210 ± 0.030	**<0.001**	**<0.001**	1.00	**<0.001**

C – control group fed with basal diet, MPPp – treated group fed a basal diet supplemented with 25 mL/day fermented with *Pediococcus pentosaceus* milk permeate; MPPa – treated group fed a basal diet supplemented with 25 mL/day fermented with *Pediococcus acidilactici* milk permeate. The data are presented as the mean ± standard deviation (*n* = 12/group). Bold values indicate significant difference between tested treatments or significant effect of the analyzed factor on tested parameters, when *p* ≤ 0.05.

### 3.4 Microbiological parameters of piglets’ faeces

Microbiological parameters of piglets’ faecal samples are shown in Table 4. Diet was a significant factor in all analysed microorganisms’ group numbers in piglets’ faeces (*p* < 0.001).

**Table 4:** Microbiological parameters of piglets’ faecal samples.

Parameter	Day	C	MPPp	MPPa	*p*			
					C × MPPp	C × MPPa	MPPp×MPPa	Significance of the analysed factor (diet)
		Microorganism count, log_10_ CFU/g						
TEC	1 day	5.81 ± 0.21^a^	7.87 ± 0.33^b^	6.42 ± 0.17^a^	<0.001	<0.001	<0.001	–
	25^th^ day	6.14 ± 0.14^b^	7.13 ± 0.24^a^	7.41 ± 0.31^b^	<0.001	<0.001	0.016	<0.001
LAB	1 day	4.22 ± 0.02^a^	4.74 ± 0.01^a^	4.33 ± 0.01^a^	<0.001	<0.001	<0.001	–
	25^th^ day	5.11 ± 0.25^b^	8.13 ± 0.25^b^	7.30 ± 0.41^b^	<0.001	<0.001	<0.001	<0.001
Y/M	1 day	4.23 ± 0.05^a^	4.93 ± 0.08^b^	3.84 ± 0.06^a^	<0.001	<0.001	<0.001	–
	25^th^ day	5.21 ± 0.21^b^	3.05 ± 0.08^a^	3.92 ± 0.10^b^	<0.001	<0.001	<0.001	<0.001

C – control group fed with basal diet, MPPp – treated group fed a basal diet supplemented with 25 mL/day fermented with *Pediococcus pentosaceus* milk permeate; MPPa – treated group fed a basal diet supplemented with 25 mL/day fermented with *Pediococcus acidilactici* milk permeate. The data are presented as the mean ± standard error (*n* = 12/group). 1 – at the beginning of the experiment; 25 – at the end of the experiment. CFU – colony-forming units; LAB – lactic acid bacteria count; TEC – total count of enterobacteria; Y/F – yeast/mould; ^a,b^ – different letters indicate significant time-related differences (*p* ≤ 0.05).

At the beginning of the experiment, the highest TEC was found in the faeces of the MP-Pp group (7.87 log_10_ CFU/g), while the daeces of the control and MPPa groups showed, on average, 26.2 and 18.4%, lower TEC, respectively. However, at the end of the experiment, in comparison to TEC in the control and both treated groups, higher TEC was established in both treated group faeces (on average, by 15.5% higher than that in the control group). At the end of the experiment, TEC showed a very strong negative correlation with faeces texture hardness (*r* = −0.832, *p* < 0.001). The MPPp group exhibited the highest LAB count, increasing from 4.74 log10 CFU/g at the start to 8.13 log10 CFU/g at the end of the experiment. At the end of the experiment, in the control group, faeces LAB count was, on average, 37.2 and 30.0%, respectively, lower than that in the MPPp and MPPa groups. A very strong positive correlation was established between TEC and LAB count at the end of the experiment (*r* = 0.827, *p* < 0.001). Also, LAB count showed a positive moderate correlation with faeces dry matter content (*r* = 0.450, *p* = 0.006) and a very strong negative correlation with faeces texture hardness (*r* = −0.849, *p* < 0.001). At the beginning of the experiment, the highest Y/M count was found in MPPp group faeces (4.93 log_10_ CFU/g). However, at the end of the experiment, this group showed the lowest number of Y/M (on average, 41.5% lower than that in the control group and, on average, 22.2% lower than that in the MP-Pa group). A very strong negative correlation was found between the Y/M and LAB counts at the end of the experiment (*r* = −0.953, *p* < 0.001). Moreover, a strong negative correlation between Y/M and TEC was established (*r* = −0.744, *p* < 0.001).

Further metataxonomic studies were performed to identify differences in the faeces’ microbial composition and their possible relations with the growth performance in different animal groups.

### 3.5 Metataxonomic composition of faecal microbiota

**Figure 4:**
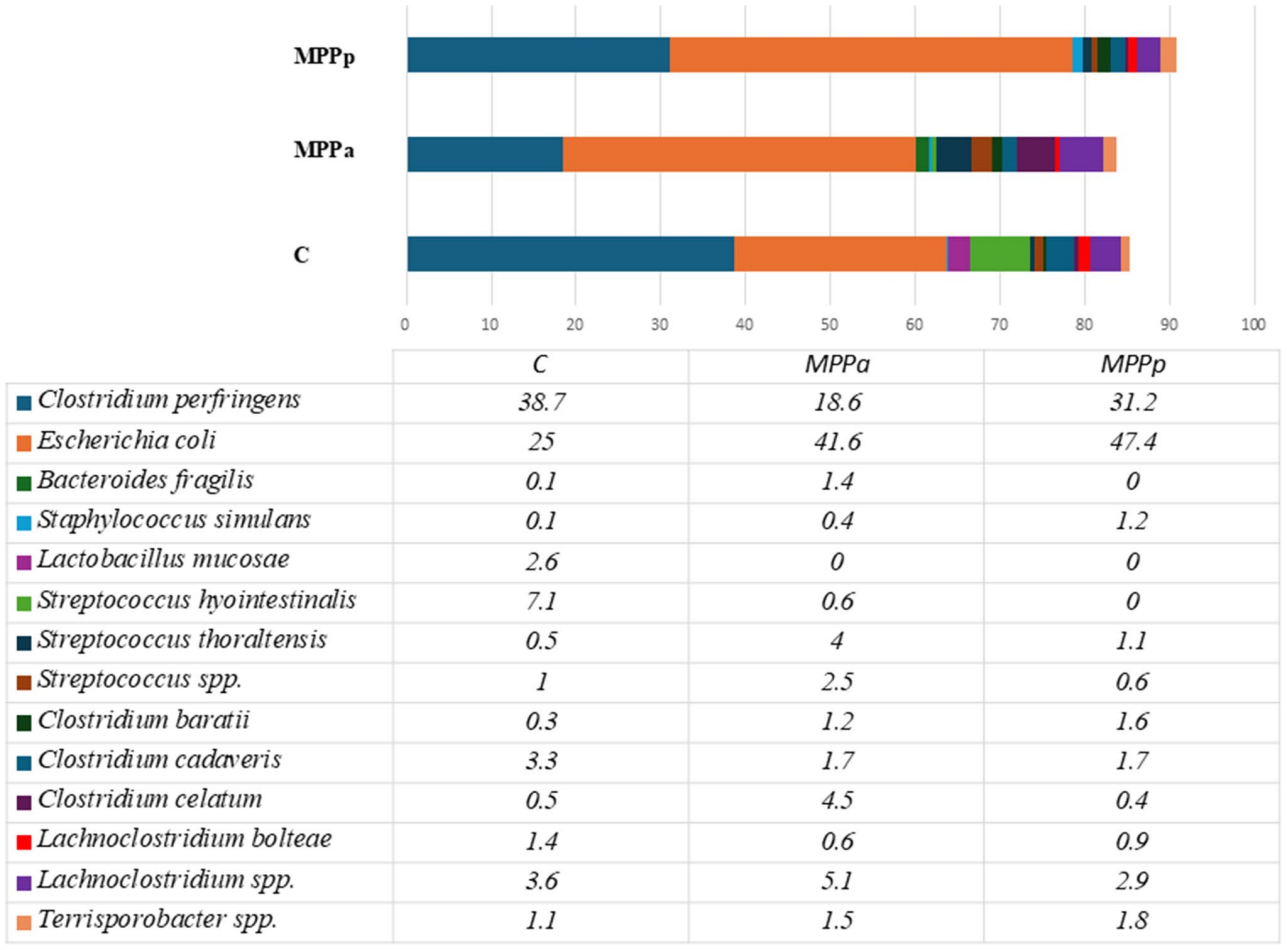
Bacteriological profiles in newborn piglet faeces from all the tested groups before experimental feeding (Only species with a prevalence of≥1% from all bacterial counts in any animal groups are presented. C – control group fed a basal diet, MPPp – treated group fed a basal diet supplemented with 25 mL/day fermented with *Pediococcus pentosaceus* milk permeate; MPPa – treated group fed a basal diet supplemented with 25 mL/day fermented with *Pediococcus acidilactici* milk permeate).

Figure 4 demonstrates bacterial composition up to the species level in 1-day-old piglet faeces before the feeding trial.

**Figure 5:**
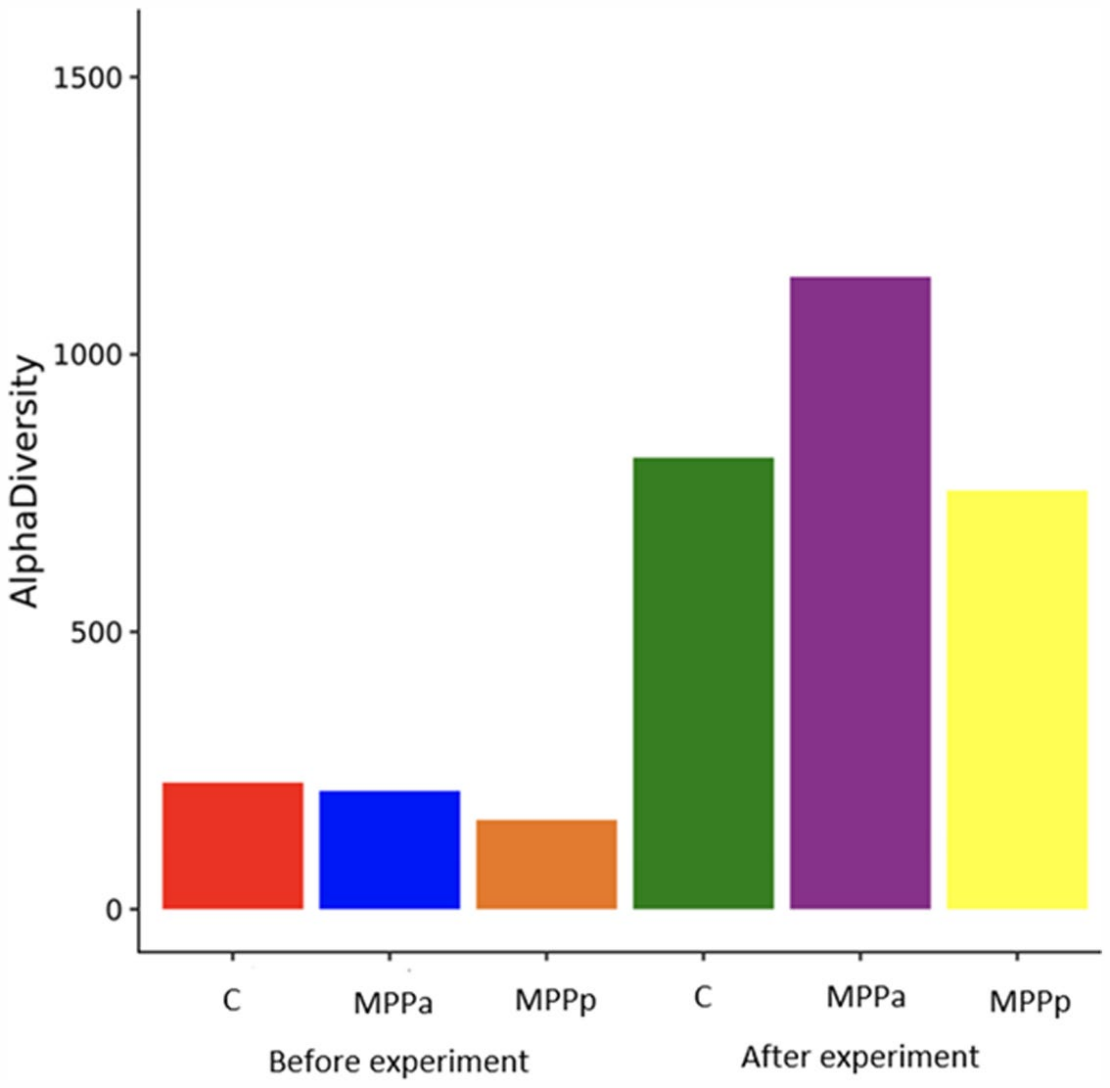
Alpha diversity of bacteria in the samples before and after the feeding experiment.

As can be seen from the data in Figure 4, two species – *Clostridium perfringens* and *Escherichia coli –* were the most prevalent in the 1st-day-old piglets, independent of the group. Those two species had a prevalence of 60.2 and 78.6%, respectively. The other most prevalent bacterial species were *Streptococcus hyointestinalis*, *Lachnoclostridium* sp., *Clostridium cadaveris*, *C. celatum*, *Streptococcus* sp., and *Terrisporobacter* sp. One species of lactobacilli (*Lactobacillus mucoasae*) had a prevalence of 2.6% among all bacteria only in one group (the control group) of the piglets, whereas there were no separate *Lactobacillus* species with a prevalence of ≥1% in the experimental groups. Overall, the *Lactobacillus* genus had a prevalence of 0.3% (MPPa group) and 2.8% (control group). Before the experiment, alpha diversity in all groups of piglets was low compared to the diversity observed after the experiment (Figure 5). At the end of the experiment, higher alpha diversity was observed in the MPPa group compared to the control and MPPp groups.

**Figure 6:**
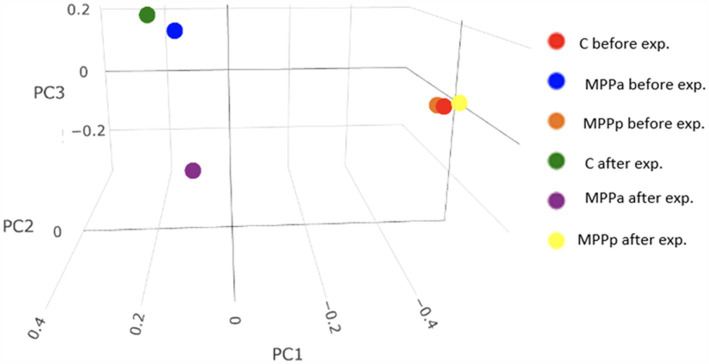
Beta diversity plot (Bray-Curtis) showing microbial diversity differences between the groups during the feeding experiment.

Microbial diversity between the groups (beta diversity) is presented in Figure 6.

Before the feeding experiment, bacterial diversity was very similar between the control and MPPp groups but rather different from the MPPa group. At the end of the experiment, beta diversity between the control group and both experimental groups demonstrated different taxonomic patterns. However, the differences between the MPPa and MPPp groups were also obvious. More detailed taxonomic composition at a genus level after the feeding experiment (day 25th) is presented in Table 5.

**Table 5:** Bacterial profiles in the pigs’ faeces on day 25 across all the tested groups at the genus level.

	Group of piglets (day 25)		
Taxonomy^a^	C	MPPa	MPPp
Methanobrevibacter**~	3.9%	3.2%	1.7%
Bacteroides**~	5.1%	3.8%	12.8%
Bacteroidales**	2.9%	4.0%	4.6%
Parabacteroides***	0.5%	1.8%	0.9%
Prevotellaceae	1.7%	2.9%	3.0%
Rikenellaceae	1.5%	1.8%	2.5%
Enterococcus***	0.3%	0.0%	1.5%
Lactobacillus**~	1.2%	2.3%	6.8%
Christensenellaceae**~	10.8%	10.6%	2.4%
Clostridium**	4.9%	3.5%	2.9%
Clostridiales I	1.0%	1.1%	1.7%
*Blautia*	1.4%	1.6%	1.3%
*Lachnoclostridium~*	2.4%	1.3%	3.2%
Marvinbryantia**	2.4%	3.3%	0.6%
Lachnospiraceae**~	6.4%	8.2%	3.4%
Clostridiales II***~	2.2%	5.1%	1.7%
*Romboutsia*	2.5%	3.5%	4.0%
Terrisporobacter**~	1.6%	2.4%	0.4%
*Anaerotruncus*	0.7%	0.9%	1.2%
Ruminococcaceae *	21.0%	15.3%	14.9%
Subdoligranulum*	5.3%	0.7%	0.7%
*Holdemanella*	0.4%	1.1%	0.8%
Erysipelotrichaceae**~	2.3%	2.1%	4.1%
*Turicibacter~*	0.6%	1.1%	0.2%
*Phascolarctobacterium*	1.9%	1.3%	1.1%
Fusobacterium*~	0.0%	0.6%	1.9%
Echerichia***~	3.1%	1.6%	3.1%
Cloacibacillus***	1.5%	0.6%	1.3%
Pyramidobacter**~	0.2%	0.2%	2.5%

*Statistically significant results (*p* ≤ 0.05) between the control and experimental groups; **Statistically significant results (*p* ≤ 0.05) between the control and MPPp group; ***Statistically significant results (*p* ≤ 0.05) between the control and MPPa group; ~ statistically significant results (*p* ≤ 0.05) between MPPa and MPPp groups.

a Higher taxonomic ranks are presented in case of undetermined genus level during identification. Only those genera are presented with a prevalence of ≥ 1% from all bacterial counts in any of the groups. C – control group fed with basal diet, MPPp – treated group fed a basal diet supplemented with 25 mL/day fermented with *Pediococcus pentosaceus* milk permeate; MPPa – treated group fed a basal diet supplemented with 25 mL/day fermented with *Pediococcus acidilactici* milk permeate.

**Figure 7:**
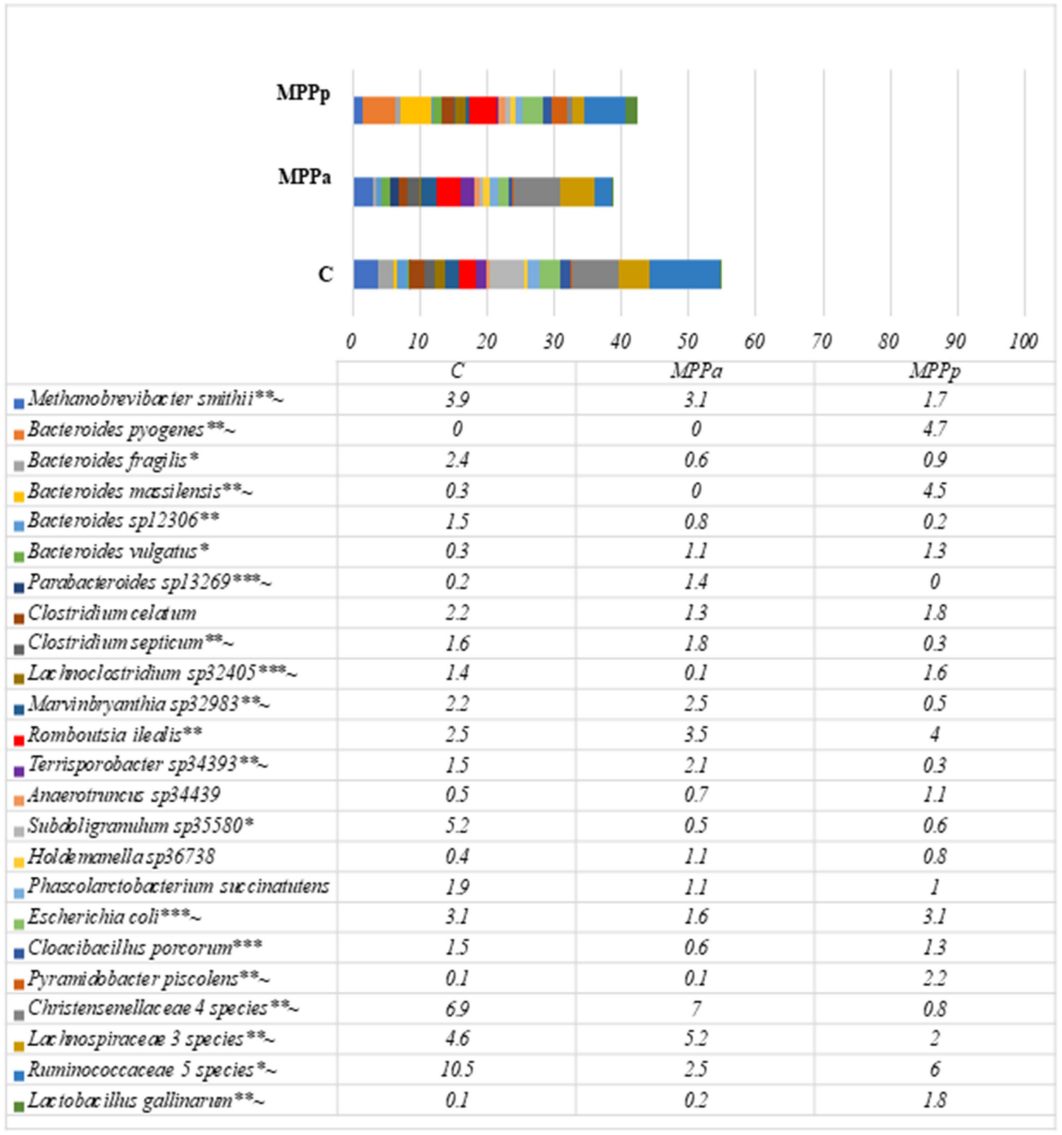
Bacterial profiles at a species level in the pigs’ faeces on day 25th (Only these species are presented, with prevalence being≥1% from all bacterial count in any animal group. C – control group fed with basal diet, MPPp – treated group fed a basal diet supplemented with 25 mL/day fermented with *Pediococcus pentosaceus* milk permeate; MPPa – treated group fed a basal diet supplemented with 25 mL/day fermented with *Pediococcus acidilactici* milk permeate). * statistically significant results (*p* ≤ 0.05) between the control and experimental groups; ** statistically significant results (*p* ≤ 0.05) between the control and MPPp group; *** statistically significant results (*p* ≤ 0.05) between the control and MPPa group ~ statistically significant results (*p* ≤ 0.05) between MPPa and MPPp groups.

On the 25th day, in all groups, the most prevalent bacteria were from the family *Ruminococcaceae* with an unestablished genus and had a prevalence from 14.9 to 21.0% from all bacterial compositions. The second most prevalent family was *Christensenellaceae,* but only in the control and MPPa groups, which had prevalences of 10.8 and 10.6%, respectively. In the MPPp group, the second most prevalent genus was *Bacteroides,* with a prevalence of 12.8%. Bacteria from this genus also had a high prevalence in other groups as well. High differences (*p* < 0.05) were detected among the amounts of *Lactobacillus*, where the highest numbers (6.8% from the total bacterial amount) were recorded in the MPPp group, whereas the prevalence of this genus was 3 and 5.7 times lower in the MPPa and C groups, respectively. When comparing bacterial genera variety in control and experimental groups, statistically significant differences were mostly detected between the control and MPPp group but not the control and MPPa group (Table 5). The most prevalent bacteria detected at a species level on day 25 are presented in Figure 7.

Metataxonomic data showed complex bacterial communities in 25-day-old piglets without clearly dominant species compared to newborn piglets; however, the overall composition differs in each animal group. Statistically significant results on bacterial prevalence at a species level between the control and both experimental groups were detected towards *Bacteroides fragilis*, *Subdoligranulum* sp., and *Ruminococcaceae* sp., with the highest prevalence of these microorganisms in the control group, whereas *Bacteroides vulgatus* has significantly lower prevalence in comparison with both experimental groups. When comparing bacterial species prevalence in the control group with separate experimental groups, the main differences were observed between the control and MPPp groups, where statistically higher prevalence was detected towards *Clostridium septicum*, *Methanobrevibacter smithii*, *Marvinbryanthia* sp., *Christensenellaceae* sp. and *Lachnobacteriaceae* sp., and the lower prevalence of *Bacteroides pyogenes*, *B. massilensis*, *Pyramidobacter piscolens,* and *Lactobacillus gallinarum* in the control group.

When comparing the MPPa and MPPp groups, statistically significant results were observed towards *Parabacteroides* sp., *Methanobrevibacter smithii*, *Clostridium septicum*, *Marvinbryanthia* sp., *Terrisporobacter* sp., *Christensenellaceae* spp., and *Lachnospiraceae* spp. with the higher prevalence in the MPPa group, whereas *Bacteroides pyogenes*, *B. massilensis*, *Lachnoclostridium* sp., *E. coli*, *Pyramidobacter piscolens*, *Ruminococcus* sp., and *Lactobacillus gallinarum* were more prevalent in the MPPp group.

### 3.6 Volatile compound profiles of piglets’ faeces

**Figure 8:**
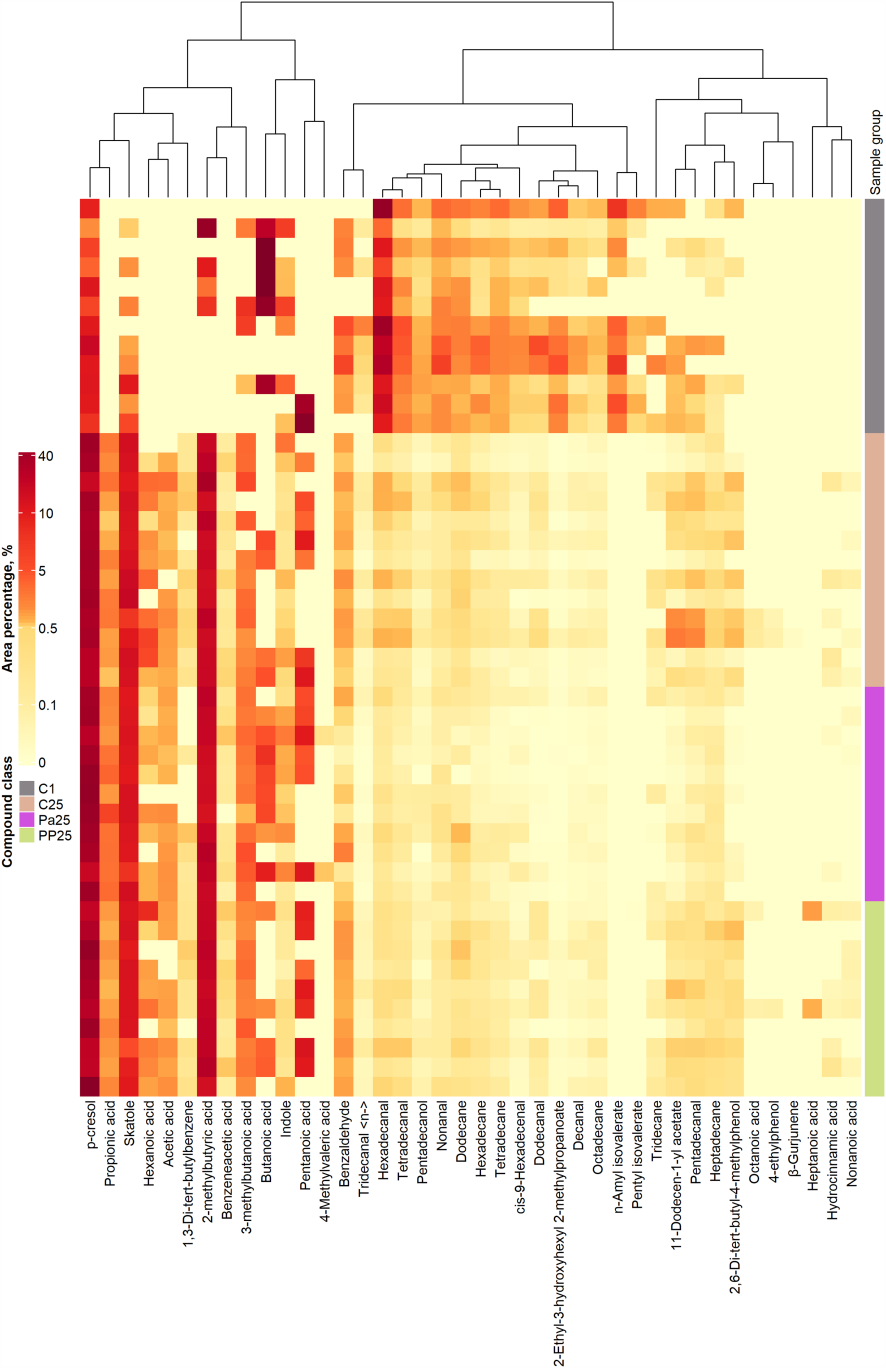
Volatile compound profiles of piglets’ faeces (a – volatile compound distribution in the control piglet groups on day 1 vs. day 25; C – control group fed a basal diet, MPPp – treated group fed a basal diet supplemented with 25 mL/day fermented with *Pediococcus pentosaceus* milk permeate; MPPa – treated group fed a basal diet supplemented with 25 mL/day fermented with *Pediococcus acidilactici* milk permeate).

**Figure 9:**
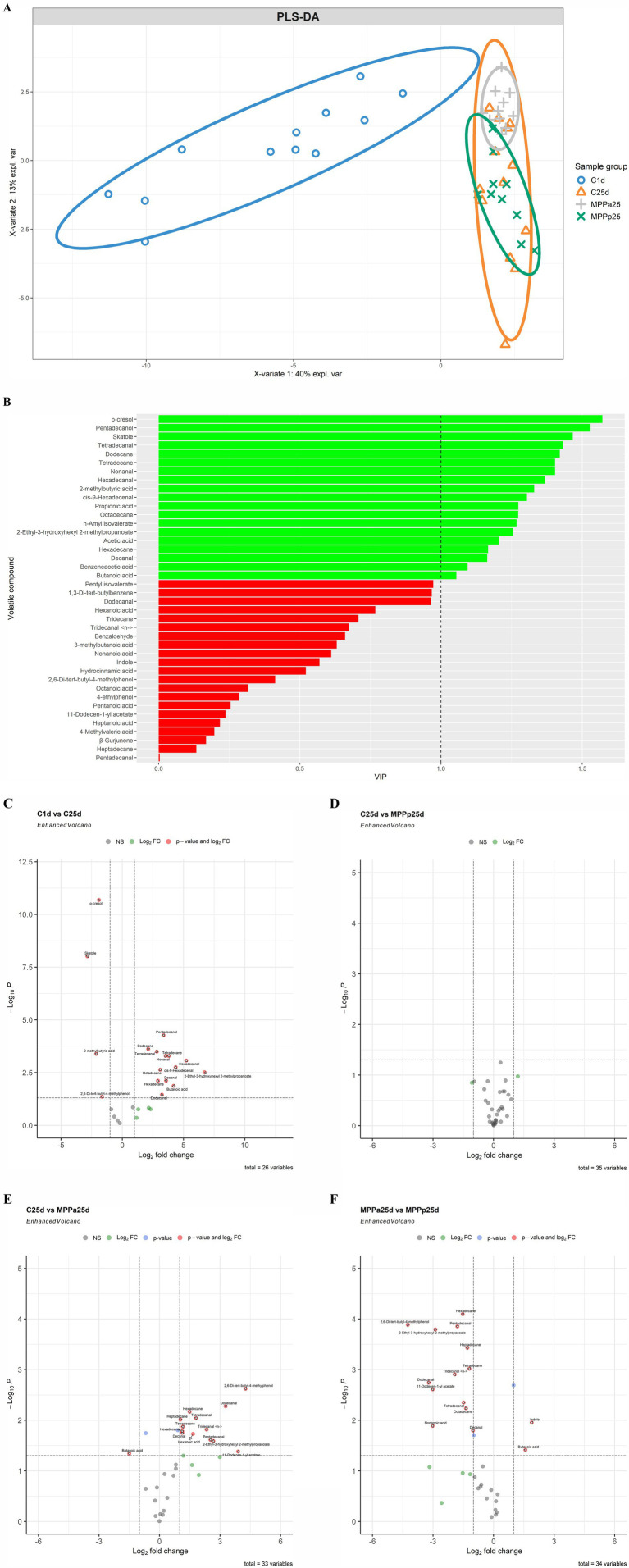
**(A)** PLS-DA plot of the detected VCs in piglet faecal sample groups. C1d, C25d – control group fed with basal diet (day 1, day 25 respectively), MPPp25 – treated group fed a basal diet supplemented with 25 mL/day fermented with *Pediococcus pentosaceus* milk permeate; MPPa25 – treated group fed a basal diet supplemented with 25 mL/day fermented with *Pediococcus acidilactici* milk permeate. **(B)** VIP score bar plot of the detected VCs in piglet faecal sample groups (Green bars denote VCs, where VIP ≥ 1, while red bars denote VCs, where VIP < 1). **(C)** Volatile compound profiles of piglets’ faeces (volatile compound distribution in the control piglets group on day 1 vs day 25; C – control group fed the basal diet, MPPp – treated group fed a basal diet supplemented with 25 mL/day fermented with *Pediococcus pentosaceus* milk permeate; MPPa – treated group fed a basal diet supplemented with 25 mL/day fermented with *Pediococcus acidilactici* milk permeate). **(D)** Volatile compound profiles of piglets’ faeces (volatile compound distribution in control *vs* MPPp groups on day 25; C – control group fed a basal diet, MPPp – treated group fed a basal diet supplemented with 25 mL/day fermented with *Pediococcus pentosaceus* milk permeate; MPPa – treated group fed a basal diet supplemented with 25 mL/day fermented with *Pediococcus acidilactici* milk permeate). **(E)** Volatile compound profiles of piglets’ faeces (volatile compound distribution in control *vs* MPPa groups on day 25; C – control group fed a basal diet, MPPp – treated group fed a basal diet supplemented with 25 mL/day fermented with *Pediococcus pentosaceus* milk permeate; MPPa – treated group fed a basal diet supplemented with 25 mL/day fermented with *Pediococcus acidilactici* milk permeate). **(F)** Volatile compound profiles of piglets’ faeces (volatile compound distribution in MPPa *vs* MPPp groups on day 25; C – control group fed with basal diet, MPPp – treated group fed a basal diet supplemented with 25 mL/day fermented with *Pediococcus pentosaceus* milk permeate; MPPa – treated group fed a basal diet supplemented with 25 mL/day fermented with *Pediococcus acidilactici* milk permeate).

The VC profiles of piglet faeces are shown in Figure 8 (VC distribution in the faeces of all piglet groups on days 1 and 25) and Figure 9 (a – PLS-DA plot of the detected VCs in piglet faecal sample groups; b – a VIP score bar plot of detected VCs in piglet faecal sample groups; c – VC distribution in control piglets on day 1 *vs* control piglets on day 25; d – VC distribution in control *vs* MPPp groups on day 25; e – VC distribution in control *vs* MPPa groups on day 25; f – VC distribution in MPPa *vs* MPPp groups on day 25).

As shown in Figure 8, a greater variety of VC in piglet faeces was identified at the end of the experiment. At the beginning of the experiment, several VCs were not detected in the faeces of all piglet groups, including acetic acid, propionic acid, 4-methylvaleric acid, hexanoic acid, heptanoic acid, 4-ethylphenol, octanoic acid, benzeneacetic acid, 1,3-di-tert-butylbenzene; non-anoic acid, hydrocinnamic acid, and *β*-gurjunene.

The main compounds in the piglet faeces VC profile included the following (which content was higher then 10% from the total VC):

1) Butanoic acid: Predominant at the beginning of the experiment, 2) 2-methylbutyric acid: At the end of the experiment, its content exceeded 20% of the total VC in all groups, 3) p-Cresol: Represented 10% of the total VC at the beginning of the experiment and increased to over 35% at the end of the experiment across all groups, 4) Skatole: Identified in all groups at the end of the experiment, and 5) Hexadecanal: Predominant at the beginning of the experiment.

PLS-DA analysis (Figure 9A) clearly separated the C1d (control group day 1) sample group from the other sample groups. Moreover, the MPPp25 (day 25) and MPPa25 (day 25) groups were notably separated. Phenolic metabolites, such as *p*-cresol in this instance, are associated with bacterial degradation of specific amino acids, such as tyrosine. The C25d (control group day 25) samples, however, exhibited much greater scattering, and their group overlapped with the aforementioned sample groups. This higher scattering in the C1d and C25d sample groups suggests higher variability in the individual VCs. In contrast, the MPPp25 and MPPa25 sample groups showed the opposite trend, suggesting a much more stable volatile profile in the faecal samples.

The VIP score analysis (Figure 9B) identified 19 analytes (VIP ≥ 1) as the most significant contributors to the variation between the sample groups. The top three VCs contributing most to group separation were *p*-cresol, pentadecanol, and skatole. Other significant VCs included various alkanes (such as tetradecane and hexadecane), aldehydes (such as decanal and tetradecanal), acids (such as acetic acid and butanoic acid), and esters (such as *n*-amyl isovalerate).

At the end of the experiment, the faecal VC profile of the control piglet group showed lower quantities of dodecanal, butanoic acid, hexadecane, decanal, 2-ethyl-3-hydroxyhexyl 2-methylpropanoate, octadecane, cis-9-hexadecenal, hexadecanal, nonanal, tetradecane, tetradecanal, dodecane, and pentadecanol (Figure 9C). In contrast, the quantities of 2-methylbutyric acid, 2,6-di-tert-butyl-4-methylphenol, skatole, and *p*-cresol were increased.

In comparison, no significant differences in individual VCs were observed between the control and MPPp groups (Figure 9D). However, when comparing the VC profiles of the control and MPPp groups with the MP-Pa group at the end of the experiment, significant differences were observed in the faecal VC profile of the MPPa (Figures 9E,F). Specifically, when comparing the control and MPPa groups, the MPPa group showed higher butanoic acid content but lower quantities of 11-dodecen-1-yl acetate, 2-ethyl-3-hydroxyhexyl 2-methylpropanoate, hexanoic acid, pentadecanal, decanal, hexadecanal, tridecanal <n->, tetradecane, heptadecane, tetradecanal, hexadecane; dodecanal, and 2,6-di-tert-butyl-4-methylphenol (Figure 9E).

In the comparison of MPPp and MPPa groups, the MPPa group exhibited higher butanoic acid and indole contents but lower quantities of decanal, nonanoic acid, octadecane, tetradecanal, 11-dodecen-1-yl acetate, dodecanal, tridecanal <n->, tetradecane, heptadecane, 2-ethyl-3-hydroxyhexyl 2-methylpropanoate, 2,6-di-tert-butyl-4-methylphenol, pentadecanal, and hexadecane (Figure 9F).

In the MPPa group, significantly lower quantities of decanal, tetradecanal, 11-dodecen-1-yl acetate, dodecanal, tridecanal <n->, tetradecane, heptadecane, 2-ethyl-3-hydroxyhexyl 2-methylpropanoate, 2,6-di-tert-butyl-4-methylphenol, pentadecanal, and hexadecane were observed compared to both the control and MPPp groups. Moreover, the MPPa group showed lower quantities of hexanoic acid and hexadecanal compared to the control group and lower quantities of nonanoic acid and octadecane compared to the MPPp group. Despite these significant differences in VC compounds between the MPPa and the other groups, further studies are warranted to clarify the metabolic pathways associated with these compounds and their relationship to the improved growth performance observed in the MPPa group.

## 4 Discussion

Newborn piglets face significant survival challenges during the first days of life, so they can benefit from the probiotic, prebiotic, and antibacterial effects of fermented milk permeate from the very beginning. The findings of this study indicate that including fermented milk permeate (MP) in piglets’ diets can positively impact their growth performance. Notably, piglets fed MP fermented with *P. acidilactici* (MP-Pa) exhibited the highest body weight throughout the experimental period, outperforming both the control group and piglets fed MP fermented with *P. pentosaceus* (MP-Pp).

This observation suggests that the specific strain of *Pediococcus* strains used for the fermentation can influence the growth-promoting effects of MP. This study applied two types of fermented MP for piglet feeding. Although both Pediococcus strains fermented MP showed similar acidity parameters, LAB numbers, and antimicrobial activities, MP fermented with *P. acidilactici* contained a higher concentration of GOS than MP fermented with *P. pentosaceus* (Supplementary material S1). The superior growth performance observed in the MPPa group might be attributed to the higher concentration of galactooligosaccharides (GOS) in this fermented MP. It was reported that GOS fed to neonatal piglets increased the number of beneficial bacteria in the intestinal microbiota and stimulated the intestinal defence mechanism (28–30). The gut microbiota of piglets is critical for nutrient digestion and can influence feed efficiency (31). However, the microbiota consists of different populations of bacteria and other microorganisms whose abundance is influenced by various factors (32). In general, the weight of piglets, from birth to weaning, is a very important parameter in commercial pig farms (33). A good pre-weaning weight can have a positive effect on piglet survival, shortening of rearing time, and rearing costs (34). Newborn piglets particularly vulnerable due to their low body weight and limited energy reserves (35). However, Ding et al. (33) reported that birth weight versus pre-weaned weight has no significant correlation, and piglets born with a higher birth weight cannot be guaranteed to gain better pre-weaning weight. Paredes et al. (36) reported that the piglets whose birth weight is not less than two standard deviations from the average population have the potential to compensate for body weight in the following phases. The feed conversion ratio is a crucial parameter of piglets and is directly related to spillage, feed digestibility, the composition of weight gain, feed intake, and nutrient utilisation (37). Interestingly, despite the differences in body weight, no significant differences were found in feed conversion ratios between the control and MPPp groups. This finding may indicate that while *P. pentosaceus* fermentation may not enhance growth to the same extent as *P. acidilactici,* it could still contribute to efficient nutrient utilisation. Due to the tight placental barriers, the newborn piglets have no antibodies (38). However, colostrum is rich in immunoglobulins (mainly IgG) (39) and growth factors (40), and to ensure passive immunity, newborn piglets receive immunoglobulin from colostrum (41). However, the absorption of immunoglobulin through the intestine only occurs up to 18 to 36 h after birth (41). On the 7th day of piglet life, total globulin concentration in the piglet’s serum can decrease (42) because maternal immunoglobulins are degraded, and new molecules can not be absorbed (41). Additionally, microbial colonisation influences the creation of the antibody profile (43, 44). In this study, diet significantly influenced IgM concentration in piglets’ plasma, and the greatest value was observed for MPPp group piglets at the end of the experiment. However, different research results have been published. It was reported that there is no significant interaction between treatment and age of piglets on IgG levels in plasma (45). Another study reported that intestinal microbiota plays an important role in the production of IgA (46). Zhao et al. (47) reported that the piglets fed with *Lactobacillus fermentum* I5007 significantly increased the amount of IgM, IgG, and IgA in the plasma of animals, as well as weaned piglets’ diet supplementation with *Lactobacillus plantarum* and fructooligosaccharides significantly increased IgG and IgA concentrations in plasma serum. Konieczka et al. (48) reported that piglets fed with *Bacillus*-based probiotic-enriched feed (during weaning) showed higher IgM levels in their plasma than in the control group. Similarly, the effects of probiotics on mammals’ plasma alanine aminotransferase and aspartate aminotransferase are heterogeneous (49). It was reported that pigs fed with *Lactobacillus acidophilus* and *Saccharomyces cerevisiae* probiotics separately and in combination were not significant on plasma alanine aminotransferase and aspartate aminotransferase concentration (50).

Increased alanine aminotransferase activity usually indicates liver cell damage, while increased aspartate aminotransferase activity may signal diseases in other organs, including the liver (51). Alanine aminotransferase and aspartate aminotransferase are located inside cells and released into the serum when the cells are broken. It was reported that the lower serum activities of alanine aminotransferase and aspartate aminotransferase in the probiotic group (supplemented with compound probiotics and berberine) indicated that antibiotics had a certain protective effect on cell function for piglets (52). In this study, diet did not significantly affect the concentration of alanine aminotransferase and aspartate aminotransferase in plasma at the end of the experiment.

For piglets to be healthy, they must have a good gastrointestinal tract barrier and function. Faecal pH measurement is a non-invasive way to assess the health of the digestive tract of piglets (53). However, the data on faecal pH measurements in pigs are scarce (54). Acid stool may indicate a digestive problem (lactose intolerance, infection, overgrowth of lactic acid bacteria) (53). It was reported that lactic acid helps to lower the pH of the sucklings’ stomachs and modify the composition of the gut microflora by lowering gut pH (55). High pH in the piglet gastrointestinal tract reduces protein digestion and increases the risk of diarrhoea in piglets during the weaning period (56). The pH of the gastrointestinal tract also depends on the composition of the feed. However, in this study, the faeces pH at the end of the experiment was similar between groups, and no effect of the diet was observed. Marchetti et al. (57) investigated that the amount of protein in the feed could have a significant effect on the composition of piglets’ faeces, and the piglets fed diets with lower protein content had higher dry matter and organic matter in their faeces. Depending on the chemical composition, but especially on solubility and microbial fermentation ability, dietary fibres can have different effects on the gastrointestinal tract and the metabolism of piglets (58). Gieryńska et al. (59) reported that insoluble dietary fibre is poorly fermentable, and its consumption increases intestinal motility and faeces volume. Pan et al. (60) found that functional oligosaccharides can significantly reduce gastrointestinal pH, improve mineral absorption, increase feed conversion efficiency, and maintain normal intestinal microflora. Palaniappan et al. (61) determine that functional oligosaccharides have the ability to form a gel consistency, bind water, reduce calories, and be used as a thickener.

Additionally, faecal texture parameters could be related to the activity of digestive enzymes (62). In our study, we observed that MP in the diet of piglets increased dry matter content in faeces and reduced their texture and hardness. It was reported that dietary fibre reduced the constipation of sows, improved gastrointestinal tract function, and softened the texture of faeces (63–66). In the first days of animal life, microbial influence is particularly important to growth, the immune system, and other health parameters (67). It was demonstrated that piglets fed with fermented feed containing a high number of *Lactobacillus* showed lower faecal *Enterobacteriaceae* counts than the control group (68). The decrease in *Enterobacteriaceae* in the faeces may be related to the ability of *Lactobacillus* to inhibit the growth of various gram-negative bacteria, especially pathogenic *E. coli*, which is well described for both *in vitro* (69) and *in vivo* conditions (70).

The provision of low pH by using acidic feed enhances the stomach barrier effect, which is the first step in defence against pathogens (71). However, *Enterobacteriaceae* can resist acidic conditions stress, which is the most frequently encountered (72). These findings can explain our study results. Also, the contribution of *Enterobacteriaceae* against colonisation of exogenous pathogens was reported (73). Additionally, *E. coli* is known to produce menaquinone (vitamin K) and vitamin B12 (74–76). Lower numbers of Y/M in the treated group piglets’ faeces can be associated with the milk permeate used in LAB antifungal activities (77). Finally, higher numbers of *Enterobacteria* and lower numbers of Y/M in the treated groups’ piglets‘faeces at the end of the experiment were not directly related to the growth performance of animals.

Recent studies by different researchers have established core microbiota in the intestinal tract of pigs. *Escherichia coli* and *Clostridium perfringens* – two predominant species found in newborn piglets in this study – are known to be the main part of the core microbiome in one-day-old piglets (78). Unterweger and colleagues tried to use these two species as probiotic therapy for the diarrhoea of neonatal piglets, meaning their physiological importance for pigs (79). Other species, including bacteria genera *Clostridium*, *Lachnoclostidium*, *Streptococcus,* and *Terrisporobacter,* were detected in all animal groups at this age.

Alpha diversity in all groups of piglets before the experiment was low. The bacterial composition and diversity of the bacterial microbiome in each animal group significantly changed on day 25th when compared with newborn piglets. Although a similar composition was detected according to taxonomic groups of bacteria in all three groups after the feeding trial, the numbers of separate taxa differed. At a genus level, microbial composition between the control and MP-Pa groups overall was similar, with only significant differences (*p* < 0.05) in the amounts of *Parabacteroides Clostridiales* II, *Enterococcus*, *Escherichia,* and *Cloacibacillus*. This demonstrates that feed supplementation with milk permeates fermented with *Pediococcus acidilactici* resulted in a less diverse variety of bacterial composition than the MPPp group. Piglets of the MPPa group also had the highest body weight gain rates compared to the control and MPPp groups. The MPPp group, however, demonstrated increased numbers in *Bacteroides* and *Lactobacillus* at a genus level, whereas *Bacteroides massilensis*, *Bacteroides pyogenes,* and *Pyramidobacter piscolens* at a species level in comparison with other groups. The MPPp group also had the lowest amounts of *Methanobrevibacter smithii*, *Parabacteroides* sp., *Clostridium septicum*, *Marvinbryanthia* sp., and some other bacterial species with still unknown importance for pigs. The amount of *Lactobacillus* in the MPPp group was almost 3 times higher than in the MPPa group, and this can be associated with the bacterial species (*Pediococcus pentosaceus*) used for the fermentation of milk permeate. At the same time, the MPPa group also had a 2 times higher amount of *Lactobacillus* than the control group.

Statistically significant differences between the groups were also observed in the higher prevalence of *Bacteroides pyogenes* and *Bacteroides massilensis* in the MPPp group, while these species were absent in the MPPa group. *Bacteroides pyogenes* is known as a pathogenic bacterium [80, 81], whereas data regarding *B. massilensis* is limited. However, it has been reported that *B. massilensis* was isolated from the blood culture of newborn babies [82]. It remains uncertain whether these species had any negative impact on the health of the piglets and if they were associated with the comparatively lower weight gain observed in the MPPp group compared to the MPPa group.

Beta diversity analysis revealed distinct bacterial taxonomic variety patterns in the faeces of the MPPp group compared to the control and MPPa groups following the experiment, suggesting that feed additives containing milk permeate fermented with *Pediococcus pentosaceus* result in more pronounced microbial changes in the pig gut compared to either the basal diet without supplementation or milk permeate fermented with *Pediococcus acidilactici*.

Faeces VC may act as a biomarker of gastrointestinal functionality because alteration in microbial and cellular metabolism could elicit variations in the VC profile of faeces (80). We observed a wider range of VCs in the piglets’ faeces at the end of the experiment. Comparative analysis of the control group’s VC profile on the 1st and 25th day of the experiment revealed a reduction in several compounds, including dodecanal, butanoic acid, hexadecane, decanal, 2-ethyl-3-hydroxyhexyl 2-methylpropanoate, octadecane, cis-9-hexadecenal, hexadecanal, nonanal, tetradecane, tetradecanal, dodecane, and pentadecanol. Conversely, the quantities of 2-methylbutyric acid, 2,6-di-tert-butyl-4-methylphenol, skatole, and p-cresol increased.

The inclusion of MPPa in the piglets’ diet induced significant changes in the VC profile compared to the control and MPPp groups. The MPPa group exhibited elevated content of butanoic acid and indole. This observation aligns with previous research indicating that galacto-oligosaccharides (GOS) in MP stimulate beneficial bacteria that produce short-chain fatty acids like butyrate (81, 82).

This production can lower colonic pH, promoting a healthier digestive tract (82, 83). While GOS may promote butyrate production by beneficial bacteria, it is important to consider that some *Clostridia* species can also produce this short-chain fatty acid (82, 83). The increase in indole within the MPPa group may be linked to microbial metabolism of dietary tryptophan (83). Indole, a bacterial signalling molecule, exhibits diverse biological activities, including antibacterial, antifungal, and immunomodulatory effects (84). However, recent studies suggest that indole may also influence bacterial persistence and antibiotic resistance (85). As significant differences in certain VCs between MPPa and other groups were obtained, further studies are needed to explain metabolic pathways, which can be related to these compounds’ formation and improved parameters of the piglets’ growth performance in the MPPa group.

According to VIP score analysis, p-cresol, pentadecanol, and skatole contributed the most to the group separation. Phenolic metabolites, such as p-cresol in this instance, are associated with bacterial degradation of specific amino acids, in this case – tyrosine and phenylalanine (86, 87). Skatole, reportedly produced by only *Clostridium* and *Lactobacillus* genera strains, is associated with the degradation of indole derivatives, in this case, the decarboxylation of indole-3-acetic acid (88). Furthermore, the aforementioned metabolites exhibit cytotoxic properties. Thus, their concentrations should be considered (86, 89). Aliphatic aldehydes can be linked to bacterial or autooxidation of unsaturated fatty acids. Therefore, their upregulation can be associated with a high-fat diet (90). Alkanes of various chain lengths can be of plant origin and get excreted in manure or faeces due to their limited absorption in the intestine or, similarly to aldehydes, can be produced by lipid peroxidation (91, 92).

## 5 Conclusion

Both types of fermented milk permeate, MPPp and MPPa, induced notable changes in the parameters tested in this study. Feed supplementation with MPPa resulted in a less diverse bacterial composition compared to the MPPp group. In total, 19 volatile compounds contributed most significantly to the variation observed between the faecel samples of the control, MPPp and MPPa groups.

MPPp feeding resulted in the greatest increase in *Lactobacillus* counts in the faeces, while MPPa feeding was associated with improved weight gain in piglets, likely due to its higher GOS concentration and distinct VC profile. Even though the growth performance of the MPPa group showed auspicious results and notable differences in VC profiles were observed between the MPPa and the other groups, further research is necessary to comprehend the metabolic pathways underlying these findings.

## Data Availability

The original contributions presented in the study are included in the article/Supplementary material, further inquiries can be directed to the corresponding author/s. Sequences were deposited at the NCBI database by the access number PRJNA1205751.

## Supplementary material

### SUPPLEMENTARY MATERIAL S1

Supplementary tables with experimental results.

### SUPPLEMENTARY TABLE S1

Acidity parameters, lactic acid bacteria count, and galactooligosaccharides content in non-fermented and 48 h fermented milk permeate.

### SUPPLEMENTARY TABLE S2

Diameter of inhibition zones (mm) in the milk permeate (MP) (0 h) and final fermented beverages (24 h), without and with addition of apple by-products, against 15 pathogenic and opportunistic bacterial strains.

### SUPPLEMENTARY TABLE S3

Antimicrobial activities of the milk permeate in liquid medium.
