# Supplementary material for: Influence of fermented milk permeate containing antimicrobial Lactobacillus and galactooligosaccharides on growth performance and health parameters in neonatal piglets
Source: Front Vet Sci. 2025 Feb 20;12:1501117. doi: 10.3389/fvets.2025.1501117 (PMC11884324; doi:10.3389/fvets.2025.1501117)
Supplement: Supplementary file 1 [file Data_Sheet_1.pdf]

Supplementary Material

Supplementary Tables

Table S1. Acidity parameters, lactic acid bacteria count, and galactooligosaccharides content in non-fermented and 48 h fermented milk permeate.

Milk Permeate	Non-fermented			Fermented 48 h				
	pH	TTA (ºN)	LAB count, $\log_{10}$ CFU/mL	pH	TTA (ºN)	LAB count, $\log_{10}$ CFU/mL	GOS, mg/100mL G2	GOS, mg/100mL G3
MPNF	5.88 ±0.8 [a]	3.0 ±0.1 [a]	nd	5.88 ±0.8 [b]	3.0 ±0.14 [a]	nd	nd	nd
MPPa	5.59 ±0.15 [a]	3.2 ±0.03 [a]	7.51 ±0.26 [a]	3.91 ±0.23 [a]	9.5 ±0.19 [b]	8.19 ±0.23 [a]	21.7 ±0.33 [b]	5.1 ±0.14
MPPp	5.61 ±0.11 [a]	3.5 ±0.2 [a]	7.16 ±0.19 [a]	3.8 ±0.15 [a]	11.0 ±0.35 [c]	8.10 ±0.22 [a]	9.1 ±0.18 [a]	nd

Supercripts [a–c] – Mean values with different letters between the lines are significantly different ($p \leq 0.05$). LAB – lactic acid bacteria; GOS – galactooligosaccharides; G2 – galactobiose; G3 – galactotriose; CFU – colony-forming units; TTA – total titratable acidity; nd – not detected. MP – milk permeate; MPPa – fermented with LUHS29 (*P. acidilactici);* MPPp – fermented with LUHS183 (*P. pentosaceus);* MPNF – non-fermented

Table S2. Diameter of inhibition zones (mm) in the milk permeate (MP) (0 h) and final fermented beverages (24 h), without and with addition of apple by-products, against 15 pathogenic and opportunistic bacterial strains.

Samples	Diameter of Inhibition zones (DIZ) (mm) Pathogenic and Opportunistic Bacterial Strains														
	1	2	3	4	5	6	7	8	9	10	11	12	13	14	15
MPPa	nd	nd	nd	nd	nd	nd	nd	nd	nd	12.7 ±0.4 [a]	nd	nd	nd	nd	15.0 ±0.1 [a]
MPPp	nd	nd	nd	nd	nd	nd	nd	nd	nd	15.4 ±0.9 [b]	nd	nd	10.3 ±0.2	nd	20.6 ±0.5 [b]
MPNF	nd	nd	nd	nd	nd	nd	nd	nd	nd	nd	nd	nd	nd	nd	nd

Supercripts[a–b] – Mean values with different letters between the lines are significantly different ($p \leq 0.05$). nd – not detected. MP – milk permeate; MPPa – fermented with LUHS29 (*P. acidilactici);* MPPp – fermented with LUHS183 (*P. pentosaceus);* MPNF - non-fermented. 1 – *Klebsiella pneumonia;* 2 – *Salmonella enterica;* 3 – *Pseudomonas aeruginosa;* 4 – *Acinetobacter baumannii;* 5 – *Proteus mirabilis;* 6 – MRSA; 7 – *Enterococcus faecalis;* 8 – *Enterococcus faecium;* 9 – *Bacillus cereus;* 10 – *Streptococcus mutans;* 11 – *Enterobacter cloacae;* 12 – *Citrobacter freundii;* 13 – *Streptococcus epidermis;* 14 – *Staphylococcus haemolyticus;* 15 – *Pasteurella multocida.*

Table S3. Antimicrobial activities of the milk permeate in liquid medium.

Samples	Growth (+) or Growth Absence (-) of Pathogenic and Opportunistic Bacteria															
	Pathogenic and Opportunistic Bacterial Strains															
	1	2	3	4	5	6	7	8	9	10	11	12	13	14	15	Number of the inhibited pathogens
Experimental design: 0.5 mL tested sample + 0.1 mL pathogen																
MPPa	+	-	-	-	-	-	+	+	-	-	-	-	-	-	-	12
MPPp	+	-	-	-	-	-	+	+	-	-	-	-	-	-	-	12
MPNF	+	+	+	+	-	-	+	+	+	+	+	+	+	+	+	0
Pathogen control	+	+	+	+	+	+	+	+	+	+	+	+	+	+	+	–
LAB control	+	+	+	+	+	+	+	+	+	+	+	+	+	+	+	–

MP – milk permeate; MPPa – fermented with LUHS29 (*P. acidilactici);* MPPp – fermented with LUHS183 (*P. pentosaceus);* MPNF – non-fermented. 1 – *Klebsiella pneumonia;* 2 – *Salmonella enterica;* 3 – *Pseudomonas aeruginosa;* 4 – *Acinetobacter baumannii;* 5 – *Proteus mirabilis;* 6 – MRSA; 7 – *Enterococcus faecalis;* 8 – *Enterococcus faecium;* 9 – *Bacillus cereus;* 10 – *Streptococcus mutans;* 11 – *Enterobacter cloacae;* 12 – *Citrobacter freundii;* 13 – *Streptococcus epidermis;* 14 – *Staphylococcus haemolyticus;* 15 – *Pasteurella multocida.*
